# Supplementary material for: A metabolomic signature of maternal BMI is associated with pregnancy complications across two independent pregnancy cohorts
Source: Commun Med (Lond). 2025 Dec 17;6:38. doi: 10.1038/s43856-025-01289-5 (PMC12820178; doi:10.1038/s43856-025-01289-5)
Supplement: Supplementary file 2 — Supplementary Information [file 43856_2025_1289_MOESM2_ESM.pdf]

SUPPLEMENTARY FIGURES

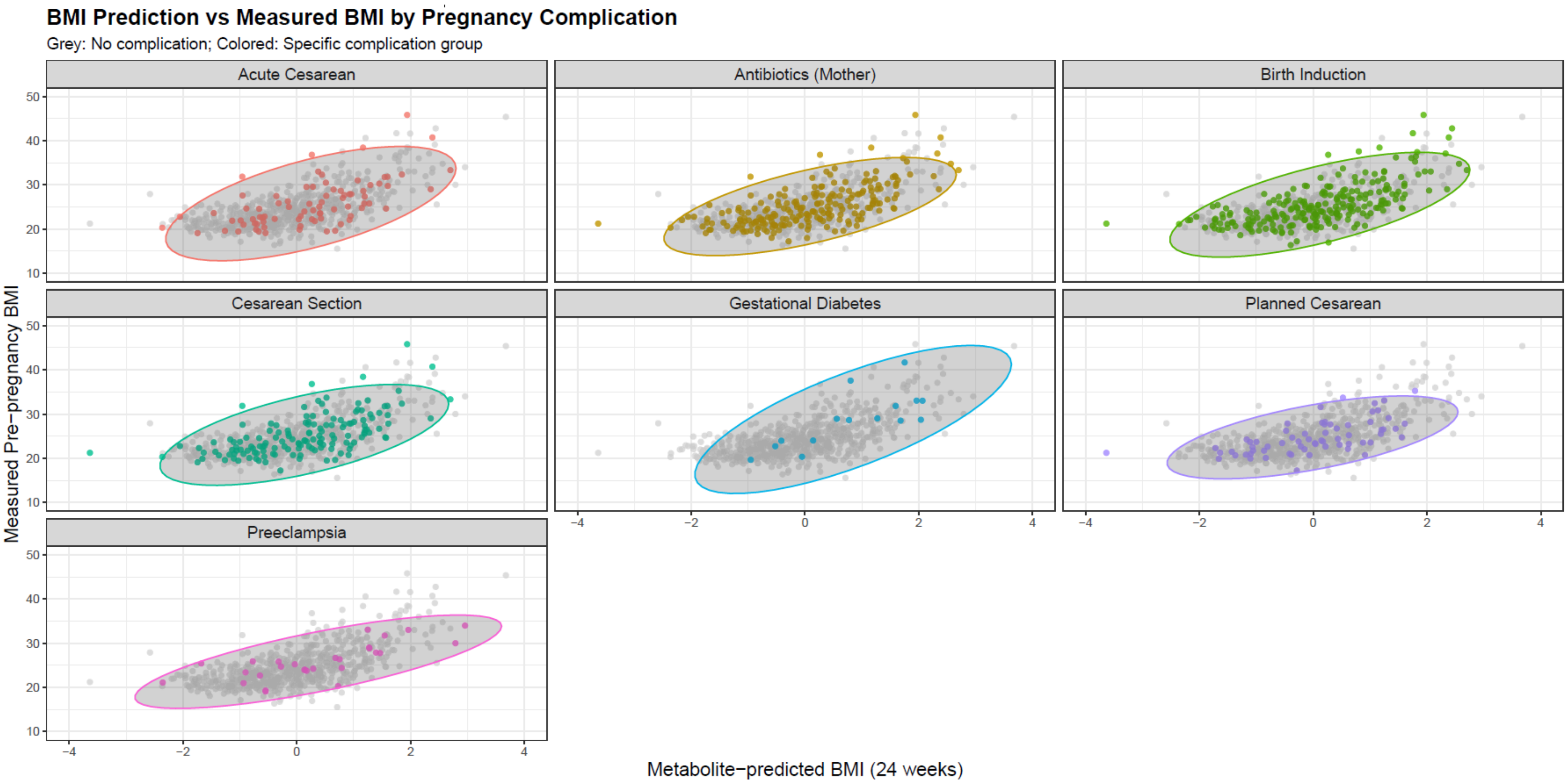

Figure S1. Relationship between Metabolite-Predicted and Measured Pre-Pregnancy BMI by Pregnancy Complication Status in COPSAC2010.

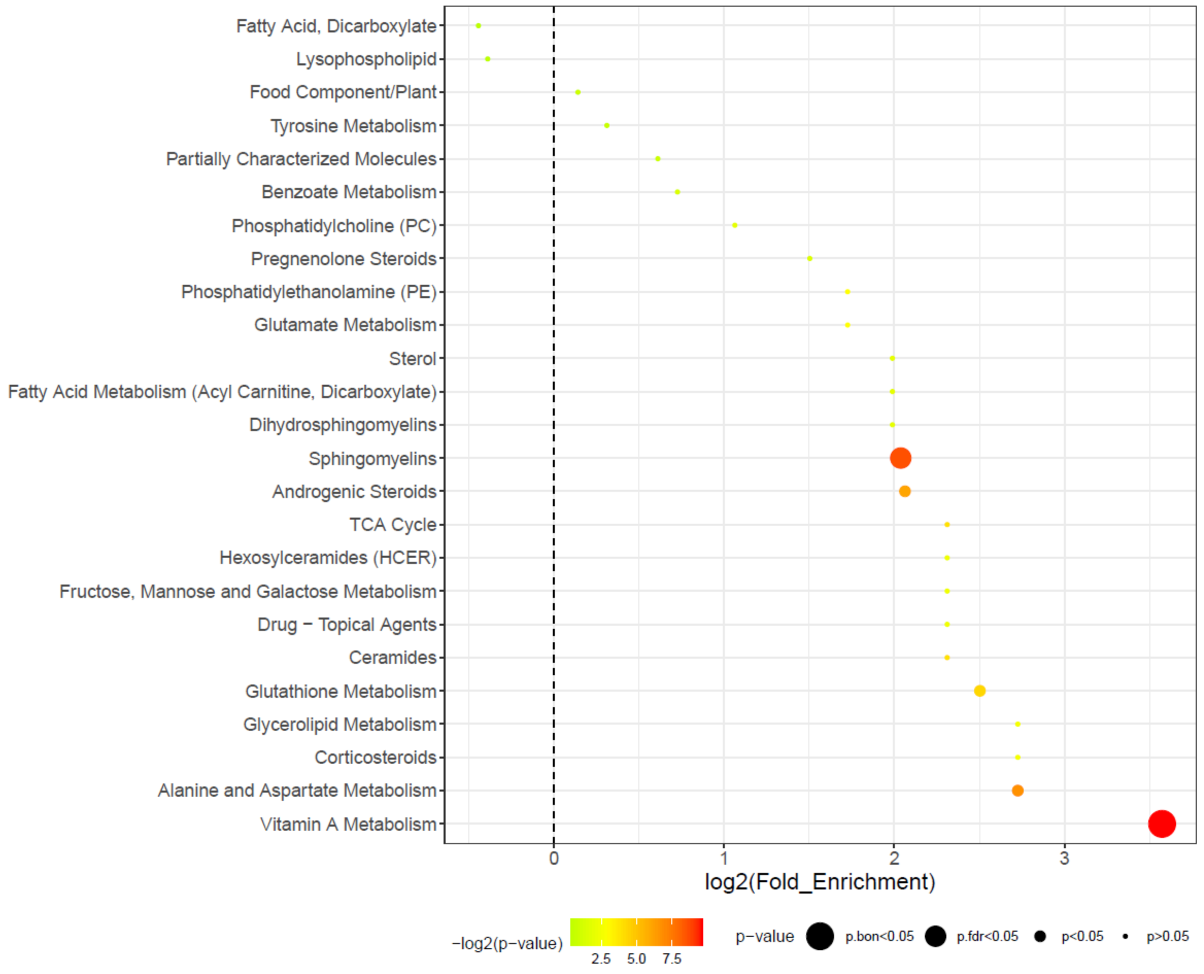

Figure S2. Pathway Enrichment Analysis of Maternal BMI-Associated Metabolites: Significant Enrichment in Sphingomyelin and Vitamin A Metabolism

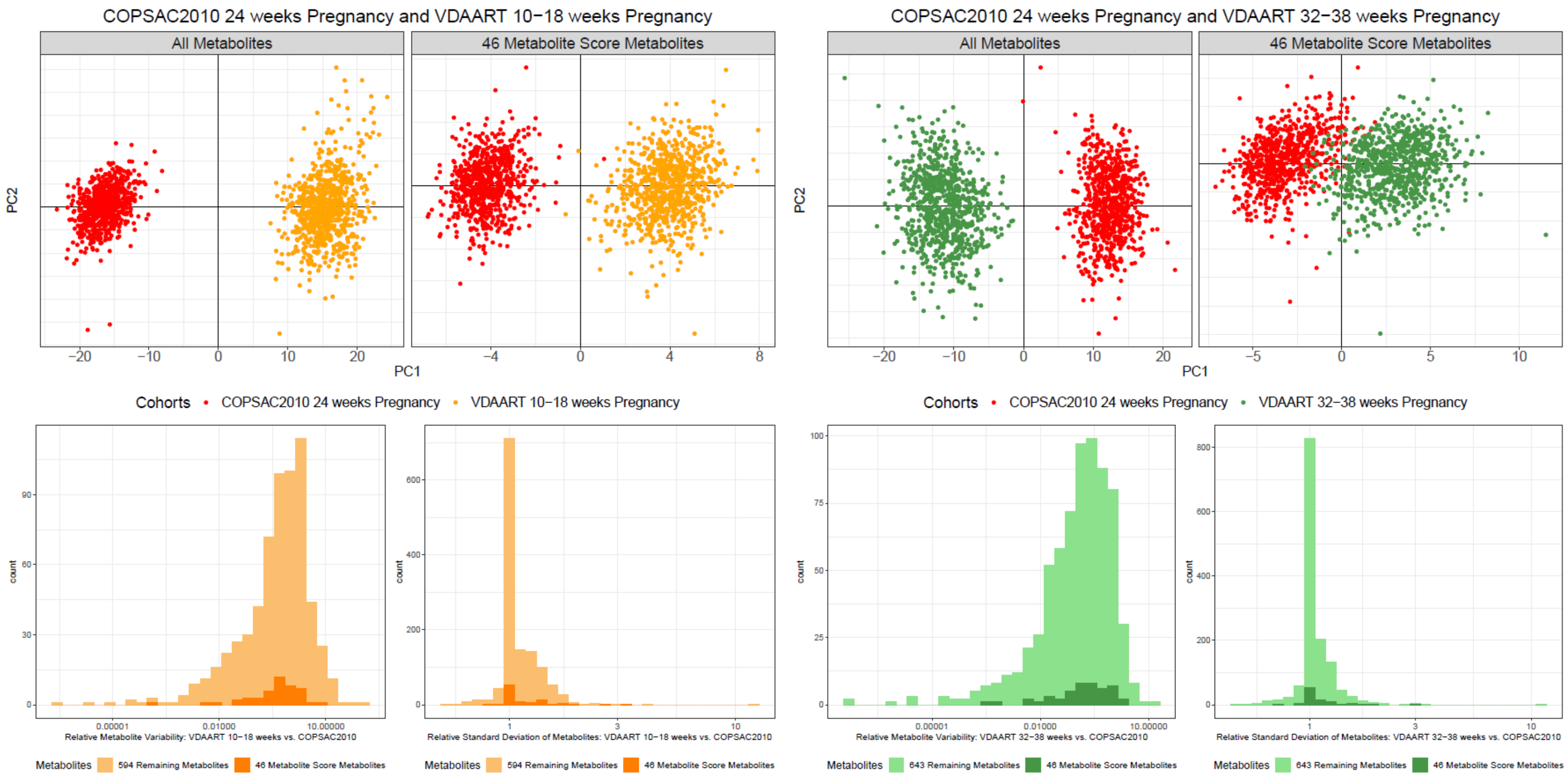
COPSAC2010 24 weeks Pregnancy and VDAART 10−18 weeks Pregnancy
All Metabolites
46 Metabolite Score Metabolites
PC2
PC1
−20
−10
0
10
20
−4
0
4
8
Cohorts
COPSAC2010 24 weeks Pregnancy
VDAART 10−18 weeks Pregnancy
COPSAC2010 24 weeks Pregnancy and VDAART 32−38 weeks Pregnancy
All Metabolites
46 Metabolite Score Metabolites
PC2
PC1
−20
−10
0
10
20
−5
0
5
10
Cohorts
COPSAC2010 24 weeks Pregnancy
VDAART 32−38 weeks Pregnancy
count
0
30
60
90
0.00001
0.01000
10.00000
Relative Metabolite Variability: VDAART 10−18 weeks vs. COPSAC2010
Metabolites
594 Remaining Metabolites
46 Metabolite Score Metabolites
count
0
200
400
600
1
3
10
Relative Standard Deviation of Metabolites: VDAART 10−18 weeks vs. COPSAC2010
Metabolites
594 Remaining Metabolites
46 Metabolite Score Metabolites
count
0
25
50
75
100
0.00001
0.01000
10.00000
Relative Metabolite Variability: VDAART 32−38 weeks vs. COPSAC2010
Metabolites
643 Remaining Metabolites
46 Metabolite Score Metabolites
count
0
200
400
600
800
1
3
10
Relative Standard Deviation of Metabolites: VDAART 32−38 weeks vs. COPSAC2010
Metabolites
643 Remaining Metabolites
46 Metabolite Score Metabolites

Figure S3. The figure compares maternal blood metabolomes at three different pregnancy timepoints from two mother-child cohorts, COPSAC2010 and VDAART. The top panels display a Principal Component Analysis (PCA) score plot for all metabolites and the selected metabolite scores comparing COPSAC2010 vs VDAART at 10-18 weeks (left) and 32-38 weeks (right) of gestation. The bottom panels illustrate the relative variation per metabolite, computed as the ratio of sums of squares (SSQ_{time} / $SSQ_{residual}$) from a one-way ANOVA model with Time/Cohort as the predictor. Additionally, it compares the per metabolite standard deviation within the cohort relative to the 24-week gestation time point from COPSAC2010, shown for COPSAC2010 vs VDAART at 10-18 weeks (left) and 32-38 weeks (right). Importantly, as modeling operates in relative space with independent centering and scaling per cohort, this comparison ensures stable variance structure of the 46 selected metabolites rather than alignment of absolute levels.

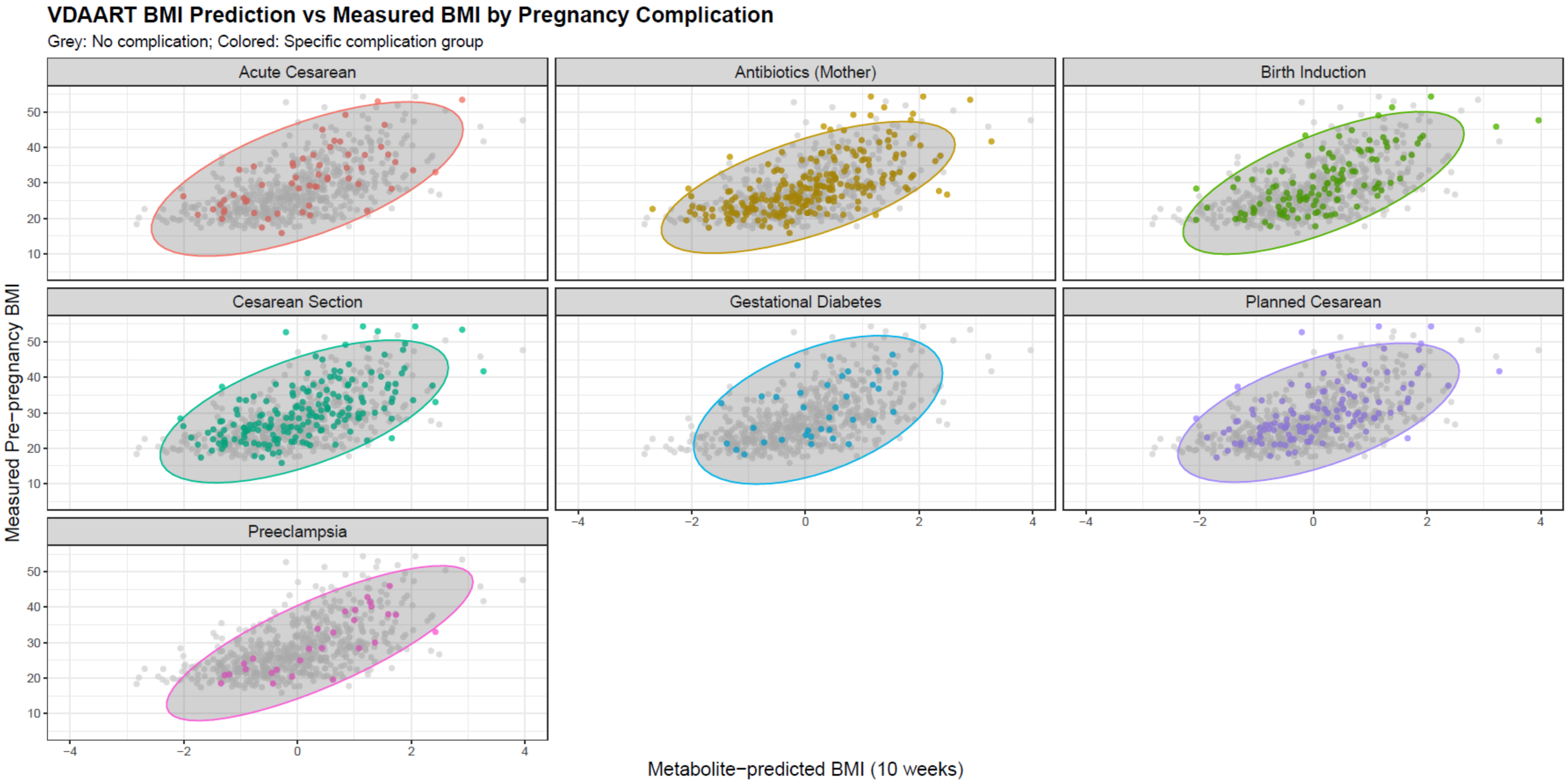

Figure S4. Relationship between maternal BMI metabolite score and measured pre-pregnancy BMI in the VDAART cohort at early gestation (10–18 weeks). Women experiencing specific pregnancy complications are highlighted against the broader cohort.

SUPPLEMENTARY TABLES

Baseline Characteristics	<18 (Underweight)	18–25 (Normal)	25–30 (Overweight)	>30 (Obese)	p-value
n =	8	436	172	74	
Male Sex (%)	3 (37.5)	224 (51.4)	92 (53.5)	34 (45.9)	0.618
White Ethnicity (%)	8 (100.0)	418 (95.9)	167 (97.1)	69 (93.2)	0.51
Income type (%)					0.848
Low	0 (0.0)	37 (14.3)	14 (12.4)	9 (15.5)	
Medium	2 (50.0)	74 (28.7)	39 (34.5)	16 (27.6)	
High	2 (50.0)	147 (57.0)	60 (53.1)	33 (56.9)	
Maternal Education Level at Birth					<0.001
Low	0 (0.0)	22 (5.0)	18 (10.5)	10 (13.5)	
Medium	5 (62.5)	267 (61.2)	107 (62.2)	59 (79.7)	
High	3 (37.5)	147 (33.7)	47 (27.3)	5 (6.8)	
Maternal age at birth (mean (SD))	31.27 (4.17)	32.31 (4.23)	32.18 (4.61)	32.25 (4.53)	0.909
Birthweight (mean (SD))	3.16 (0.48)	3.50 (0.55)	3.64 (0.52)	3.65 (0.48)	0.001
Gestational age (mean (SD))	273.38 (14.13)	278.79 (11.70)	280.51 (11.49)	279.47 (8.62)	0.175
Cesarean section (%)	1 (12.5)	83 (19.0)	43 (25.0)	21 (28.4)	0.152
Maternal smoking during pregnancy (%)	1 (12.5)	31 (7.1)	16 (9.3)	6 (8.1)	0.782
Siblings (mean (SD))	1.25 (0.46)	1.54 (0.78)	1.57 (0.79)	1.69 (1.34)	0.441
Pregnancy Varied Dietary Pattern (mean (SD))	0.59 (0.78)	0.07 (0.99)	-0.11 (0.93)	-0.05 (1.20)	0.135
Pregnancy Western Dietary Pattern (mean (SD))	0.53 (0.98)	-0.08 (1.02)	0.01 (0.88)	0.30 (1.06)	0.02
Pregnancy PC3 Dietary Pattern (mean (SD))	0.29 (0.50)	0.08 (1.00)	-0.04 (0.98)	-0.33 (0.98)	0.02
Maternal Pre-pregnancy BMI (mean (SD))	16.99 (0.81)	22.11 (1.66)	27.00 (1.42)	34.05 (3.72)	<0.001
Maternal BMI Metabolite Score (mean (SD))	-0.19 (0.53)	-0.44 (0.79)	0.40 (0.80)	1.39 (0.81)	<0.001

Table S1. Baseline characteristics stratified by clinical maternal pre-pregnancy BMI categories (underweight, normal weight, overweight, and obese) among pregnant mothers in the COPSAC2010 cohort. Group comparisons use two-sided Pearson's χ^2 tests for categorical variables and two-sided one-way ANOVA for continuous variables.

Cohort Characteristics	COPSAC2010	VDAART	p-value
n =	684	775	
Male Sex (%)	350 (51.2)	402 (51.9)	0.81
White Ethnicity (%)	655 (95.8)	304 (40.9)	<0.001
Income type (%)			<0.001
Low	58 (13.6)	97 (35.8)	
Medium	128 (30.0)	93 (34.3)	
High	240 (56.3)	81 (29.9)	
Maternal Education Attainment at Birth			<0.001
Low	50 (7.3)	285 (38.4)	
Medium	434 (63.5)	204 (27.5)	
High	200 (29.2)	254 (34.2)	
Maternal age at birth (years) (mean (SD))	32.30 (4.36)	25.39 (5.47)	<0.001
Maternal Pre-pregnancy BMI (mean(SD))	24.56 (4.41)	28.38 (7.79)	<0.001
Maternal Pre-pregnancy BMI Obesity Category			
<18 (Underweight)	8 (1.2)	11 (1.7)	<0.001
18–25 (Normal)	428 (63.0)	235 (36.8)	
25–30 (Overweight)	170 (25.0)	188 (29.4)	
>30 (Obese)	73 (10.8)	205 (32.1)	
Birthweight (kilograms) (mean (SD))	3.55 (0.54)	3.27 (0.57)	<0.001
Gestational age (days) (mean (SD))	279.28 (11.30)	272.96 (13.85)	<0.001
Maternal smoking during pregnancy (%)	52 (7.6)	18 (2.3)	<0.001
Gestational Diabetes (%)	15 (2.2)	40 (5.2)	0.005
Preeclampsia (%)	29 (4.2)	34 (4.4)	0.981
C-section (%)	148 (21.6)	229 (29.5)	0.001
Acute C-section (%)	83 (12.1)	70 (9.0)	0.065
Elective C-section (%)	65 (9.5)	159 (20.5)	<0.001
Induction of Birth (%)	243 (35.6)	141 (18.2)	<0.001
Maternal Antibiotics at Birth (%)	219 (32.2)	299 (38.7)	0.011

Table S2. Characteristics of participants in the COPSAC2010 and VDAART cohorts. P-values indicate significant differences between the two cohorts. The income categories are defined as <€50,000, €50,000 - €110,000, and >€110,000 in COPSAC2010, and <$30,000, $30,000 - $99,999, and >$100,000 USD in VDAART. Educational attainment levels are categorised as “primary, secondary, or college graduate”, “tradesman or bachelor's degree” and “Master's degree” in COPSAC2010, and “Did not graduate from high school/Graduated from high school”, “Technical school/Junior college/some college” and “Graduate school/College graduate” in VDAART. Group comparisons use two-sided Pearson’s χ^2 tests for categorical variables and two-sided one-way ANOVA for continuous variables.

BIOCHEMICAL	SUPER PATHWAY	SUB PATHWAY	Comp.1	Gestation Diabetes Mediating Metabolite
cortolone glucuronide (1)	Lipid	Corticosteroids	2.935 x 10^-3	Yes
4-hydroxyglutamate	Amino Acid	Glutamate Metabolism	2.364 x 10^-3	Yes
glutamate	Amino Acid	Glutamate Metabolism	2.330 x 10^-3	No
1-stearoyl-2-arachidonoyl-GPE (18:0/20:4)	Lipid	Phosphatidylethanolamine (PE)	2.318 x 10^-3	No
glycosyl-N-(2-hydroxynervonoyl)-sphingosine (d18:1/24:1(2OH))*	Lipid	Hexosylceramides (HCER)	2.271 x 10^-3	No
glycerol	Lipid	Glycerolipid Metabolism	2.260 x 10^-3	Yes
sphingomyelin (d18:2/16:0, d18:1/16:1)*	Lipid	Sphingomyelins	2.259 x 10^-3	No
sphingomyelin (d18:0/18:0, d19:0/17:0)*	Lipid	Dihydrosphingomyelins	2.220 x 10^-3	No
sphingomyelin (d18:1/18:1, d18:2/18:0)	Lipid	Sphingomyelins	2.161 x 10^-3	No
N-stearoyl-sphingosine (d18:1/18:0)*	Lipid	Ceramides	2.144 x 10^-3	No
1-palmitoyl-2-arachidonoyl-GPC (16:0/20:4n6)	Lipid	Phosphatidylcholine (PC)	2.124 x 10^-3	No
1-stearoyl-2-arachidonoyl-GPC (18:0/20:4)	Lipid	Phosphatidylcholine (PC)	2.121 x 10^-3	No
hydroxyasparagine	Amino Acid	Alanine and Aspartate Metabolism	2.098 x 10^-3	No
sphingomyelin (d17:2/16:0, d18:2/15:0)*	Lipid	Sphingomyelins	2.034 x 10^-3	Yes
sphingomyelin (d18:2/21:0, d16:2/23:0)*	Lipid	Sphingomyelins	1.998 x 10^-3	No
sphingomyelin (d18:2/14:0, d18:1/14:1)*	Lipid	Sphingomyelins	1.978 x 10^-3	No
sphingomyelin (d18:2/18:1)*	Lipid	Sphingomyelins	1.943 x 10^-3	No
carotene diol (1)	Cofactors and Vitamins	Vitamin A Metabolism	-1.927 x 10^-3	Yes
16a-hydroxy DHEA 3-sulfate	Lipid	Androgenic Steroids	1.910 x 10^-3	No
pregnenetriol disulfate*	Lipid	Pregnenolone Steroids	1.901 x 10^-3	No

BIOCHEMICAL	SUPER PATHWAY	SUB PATHWAY	Comp.1	Gestation Diabetes Mediating Metabolite
asparagine	Amino Acid	Alanine and Aspartate Metabolism	-1.898 x 10^-3	No
3-phenylpropionate (hydrocinnamate)	Xenobiotics	Benzoate Metabolism	-1.869 x 10^-3	No
mannose	Carbohydrate	Fructose, Mannose and Galactose Metabolism	1.839 x 10^-3	Yes
androstenediol (3beta,17beta) disulfate (1)	Lipid	Androgenic Steroids	1.807 x 10^-3	No
ceramide (d18:2/24:1, d18:1/24:2)*	Lipid	Ceramides	1.757 x 10^-3	Yes
beta-cryptoxanthin	Cofactors and Vitamins	Vitamin A Metabolism	-1.746 x 10^-3	No
tartronate (hydroxymalonate)	Xenobiotics	Food Component/Plant	-1.703 x 10^-3	Yes
alpha-ketoglutarate	Energy	TCA Cycle	1.696 x 10^-3	Yes
andro steroid monosulfate C19H28O6S (1)*	Lipid	Androgenic Steroids	1.680 x 10^-3	Yes
carotene diol (2)	Cofactors and Vitamins	Vitamin A Metabolism	-1.673 x 10^-3	Yes
cysteinylglycine disulfide*	Amino Acid	Glutathione Metabolism	1.657 x 10^-3	No
branched chain 14:0 dicarboxylic acid	Lipid	Fatty Acid, Dicarboxylate	-1.655 x 10^-3	Yes
gentisate	Amino Acid	Tyrosine Metabolism	-1.598 x 10^-3	Yes
adipoylcarnitine (C6-DC)	Lipid	Fatty Acid Metabolism (Acyl Carnitine, Dicarboxylate)	1.594 x 10^-3	No
2,6-dihydroxybenzoic acid	Xenobiotics	Drug - Topical Agents	-1.586 x 10^-3	No
mannonate*	Xenobiotics	Food Component/Plant	1.581 x 10^-3	No
aspartate	Amino Acid	Alanine and Aspartate Metabolism	1.572 x 10^-3	No

BIOCHEMICAL	SUPER PATHWAY	SUB PATHWAY	Comp.1	Gestation Diabetes Mediating Metabolite
androstenediol (3beta,17beta) disulfate (2)	Lipid	Androgenic Steroids	1.567 x 10^-3	No
metabolonic lactone sulfate	Partially Characterized Molecules	Partially Characterized Molecules	1.565 x 10^-3	No
1-palmitoyl-2-arachidonoyl-GPE (16:0/20:4)*	Lipid	Phosphatidylethanolamine (PE)	1.528 x 10^-3	No
4-ethylphenyl sulfate	Xenobiotics	Benzoate Metabolism	-1.512 x 10^-3	No
aconitate [cis or trans]	Energy	TCA Cycle	1.483 x 10^-3	Yes
3beta-hydroxy-5-cholestenoate	Lipid	Sterol	-1.480 x 10^-3	Yes
1-palmitoleoyl-GPC* (16:1)*	Lipid	Lysophospholipid	1.479 x 10^-3	No
ergothioneine	Xenobiotics	Food Component/Plant	-1.473 x 10^-3	No
cys-gly, oxidized	Amino Acid	Glutathione Metabolism	1.472 x 10^-3	Yes

Table S3. The 46 metabolites selected by the sparse partial least squares model regularisation with maternal pre-pregnancy BMI as the response in COPSAC2010 (overlap between metabolites at 10-18 weeks timepoint in VDAART) . Metabolites name, super pathway, sub pathways,loadings for component 1 and whether or not the metabolite significantly mediated the association between the maternal pre-pregnancy BMI and Gestational Diabetes.

	Missingness all metabolites (#1131)		Missingness selected metabolites (#46)	
**Pregnancy Complications**	**Odds Ratio [95% CI] p-value**	**p-value**	**Odds Ratio [95% CI]**	**p-value**
**Gestational Diabetes**	OR 0.97 [0.92; 1.03]	0.381	OR 1.57 [0.86; 2.55]	0.099
**Preeclampsia**	OR 0.98 [0.93; 1.02]	0.241	OR 1.03 [0.57; 1.65]	0.899
**Caesarean Section**	OR 1.00 [0.98; 1.02]	0.995	OR 0.73 [0.52; 0.97]	0.04
**Emergency**	OR 0.99 [0.97; 1.02]	0.472	OR 0.80 [0.53; 1.13]	0.238
**Elective**	OR 1.01 [0.98; 1.04]	0.418	OR 0.69 [0.41; 1.05]	0.115
**Induction of Birth**	OR 1.01 [1.00; 1.03]	0.173	OR 0.93 [0.74; 1.17]	0.541
**Maternal Antibiotics at Birth**	OR 1.0 [0.99; 1.02]	0.698	OR 0.91 [0.71; 1.15]	0.431
**Exposure**	**Estimate [95% CI]**	**p-value**	**Estimate [95% CI]**	**p-value**
**Maternal Pre-pregnancy BMI**	0.00 [0.00; 0.01]	0.61	0.04 [-0.15; 0.07]	0.51

Table S4. Analysis of missing metabolites and their association with maternal pre-pregnancy BMI. Missingness rates of all metabolites and the selected 46 metabolites in the maternal BMI metabolite score, alongside their odds ratios and confidence intervals for pregnancy complication outcomes and pre-pregnancy BMI. P-values are from two-sided Wald tests. No adjustments were made for multiple comparisons.

COPSAC2010 Pregnancy Complications	Maternal BMI (per unit change)
**Gestational Diabetes**	1.16 [1.06 - 1.26], $p = 6.75 \times 10^{-4}$)
**Preeclampsia**	1.05 [0.97 - 1.12], p = 0.237)
**Cesarean Section**	1.05 [1.01 - 1.09], p = 0.023)
**Emergency**	1.06 [1.01 - 1.11], p = 0.015)
**Elective**	1.01 [0.95 - 1.07], p = 0.628)
**Induction of Birth**	1.08 [1.04 - 1.12], $p = 2.86 \times 10^{-5}$)
**Antibiotics Administered to Mother at Birth**	1.04 [1 - 1.08], p = 0.042)
**VDAART Pregnancy Complications**	**Maternal BMI (per unit change)**
**Gestational Diabetes**	1.05 [1.01 - 1.1], p = 0.012)
**Preeclampsia**	1.01 [0.97 - 1.06], p = 0.553)
**Cesarean Section**	1.04 [1.02 - 1.06], $p = 5.60 \times 10^{-4}$)
**Emergency**	1.04 [1.01 - 1.07], p = 0.014)
**Elective**	1.03 [1 - 1.05], p = 0.033)
**Induction of Birth**	1.03 [1.01 - 1.06], p = 0.008)
**Antibiotics Administered to Mother at Birth**	1.01 [0.99 - 1.04], p = 0.196)

Table S5. Results of the association between maternal pre-pregnancy BMI and pregnancy complications, per unit change of BMI, in multivariable modelling. P-values are from two-sided Wald tests. No adjustments were made for multiple comparisons.

Maternal BMI Associated Metabolites in VDAART	Pre-eclampsia		Gestational Diabetes	
	10-18 Weeks	32-38 Weeks	10-18 Weeks	32-38 Weeks
16a-hydroxy DHEA 3-sulfate	1.02 [0.72 - 1.46], p = 0.900	1.16 [0.8 - 1.67], p = 0.434	1.36 [0.97 - 1.91], p = 0.073	1.49 [1.07 - 2.08], p = 0.019
**1-lignoceroyl-GPC (24:0)**	-	1.05 [0.72 - 1.52], p = 0.802	-	0.45 [0.32 - 0.63], p = <0.001
1-palmitoleoyl-GPC* (16:1)*	1.23 [0.86 - 1.77], p = 0.256	1.11 [0.77 - 1.61], p = 0.577	0.96 [0.69 - 1.36], p = 0.835	0.58 [0.41 - 0.81], p = 0.001
1-palmitoyl-2-arachidonoyl-GPC (16:0/20:4n6)	1.08 [0.76 - 1.55], p = 0.654	0.91 [0.63 - 1.34], p = 0.642	1.2 [0.87 - 1.66], p = 0.283	1.21 [0.88 - 1.68], p = 0.258
1-palmitoyl-2-arachidonoyl-GPE (16:0/20:4)*	1.38 [0.97 - 1.98], p = 0.079	1.45 [1 - 2.14], p = 0.054	1.28 [0.92 - 1.79], p = 0.144	1.65 [1.17 - 2.36], p = 0.005
1-stearoyl-2-arachidonoyl-GPC (18:0/20:4)	0.9 [0.63 - 1.3], p = 0.559	0.96 [0.66 - 1.43], p = 0.849	1.07 [0.78 - 1.49], p = 0.671	1.26 [0.9 - 1.79], p = 0.178
1-stearoyl-2-arachidonoyl-GPE (18:0/20:4)	1.28 [0.89 - 1.86], p = 0.194	1.13 [0.78 - 1.68], p = 0.524	1.41 [1.01 - 2], p = 0.051	2.49 [1.72 - 3.68], p = <0.001
2,6-dihydroxybenzoic acid	0.85 [0.58 - 1.24], p = 0.395	1.03 [0.67 - 1.57], p = 0.907	0.97 [0.67 - 1.41], p = 0.878	0.81 [0.54 - 1.22], p = 0.318
3beta-hydroxy-5-cholestenoate	1.06 [0.75 - 1.5], p = 0.735	0.99 [0.68 - 1.44], p = 0.962	0.53 [0.37 - 0.73], p = <0.001	0.66 [0.47 - 0.92], p = 0.015
3-phenylpropionate (hydrocinnamate)	1.07 [0.74 - 1.56], p = 0.734	0.88 [0.6 - 1.29], p = 0.496	0.89 [0.65 - 1.22], p = 0.456	0.72 [0.53 - 1], p = 0.045
4-ethylphenyl sulfate	0.8 [0.54 - 1.16], p = 0.261	1.11 [0.75 - 1.61], p = 0.592	1.08 [0.78 - 1.46], p = 0.635	1 [0.73 - 1.35], p = 0.988
4-hydroxyglutamate	1.17 [0.83 - 1.65], p = 0.377	2.05 [1.41 - 3.01], p = <0.001	2.01 [1.43 - 2.82], p = <0.001	1.75 [1.25 - 2.48], p = 0.001
aconitate [cis or trans]	1.1 [0.75 - 1.77], p = 0.676	1.49 [0.94 - 2.44], p = 0.109	1.75 [1.1 - 2.84], p = 0.020	1.88 [1.23 - 2.91], p = 0.004
adipoylcarnitine (C6-DC)	1.32 [0.95 - 1.84], p = 0.097	1.51 [1.06 - 2.14], p = 0.020	0.98 [0.7 - 1.36], p = 0.889	1.8 [1.33 - 2.45], p = <0.001
alpha-ketoglutarate	1.05 [0.76 - 1.49], p = 0.770	1.11 [0.77 - 1.56], p = 0.570	1.02 [0.74 - 1.45], p = 0.910	0.98 [0.7 - 1.37], p = 0.922

Maternal BMI Associated Metabolites in VDAART	Pre-eclampsia		Gestational Diabetes	
	**10-18 Weeks**	**32-38 Weeks**	**10-18 Weeks**	**32-38 Weeks**
andro steroid monosulfate C19H28O6S (1)*	1.1 [0.77 - 1.57], p = 0.594	1.36 [0.94 - 2], p = 0.107	1.28 [0.91 - 1.79], p = 0.157	1.32 [0.93 - 1.86], p = 0.117
androstenediol (3beta,17beta) disulfate (1)	1.1 [0.78 - 1.57], p = 0.581	1.25 [0.86 - 1.83], p = 0.254	2.02 [1.43 - 2.89], p = <0.001	1.17 [0.84 - 1.63], p = 0.363
androstenediol (3beta,17beta) disulfate (2)	1.09 [0.77 - 1.54], p = 0.617	1.07 [0.74 - 1.56], p = 0.716	1.57 [1.14 - 2.2], p = 0.005	1.23 [0.89 - 1.7], p = 0.199
asparagine	0.93 [0.75 - 1.32], p = 0.604	0.74 [0.49 - 1.09], p = 0.131	0.95 [0.75 - 1.37], p = 0.724	0.58 [0.4 - 0.82], p = 0.003
aspartate	1.03 [0.71 - 1.46], p = 0.882	1.17 [0.8 - 1.67], p = 0.403	1.14 [0.83 - 1.57], p = 0.409	1.5 [1.09 - 2.06], p = 0.012
beta-cryptoxanthin	0.78 [0.57 - 1.1], p = 0.149	0.66 [0.46 - 0.97], p = 0.033	0.64 [0.48 - 0.87], p = 0.003	0.64 [0.47 - 0.88], p = 0.006
branched chain 14:0 dicarboxylic acid	0.97 [0.68 - 1.4], p = 0.875	0.87 [0.58 - 1.28], p = 0.486	0.87 [0.62 - 1.21], p = 0.395	0.71 [0.5 - 1], p = 0.055
carotene diol (1)	0.79 [0.57 - 1.12], p = 0.163	0.64 [0.46 - 0.92], p = 0.011	0.91 [0.67 - 1.26], p = 0.549	0.96 [0.7 - 1.34], p = 0.811
carotene diol (2)	0.86 [0.62 - 1.23], p = 0.412	0.71 [0.5 - 1.03], p = 0.066	0.91 [0.67 - 1.24], p = 0.535	0.88 [0.65 - 1.21], p = 0.429
**ceramide (d18:2/24:1, d18:1/24:2)***	0.99 [0.69 - 1.4], p = 0.951	-	0.84 [0.61 - 1.16], p = 0.281	-
cortolone glucuronide (1)	1.24 [0.88 - 1.79], p = 0.236	1.24 [0.86 - 1.82], p = 0.255	1.7 [1.19 - 2.48], p = 0.004	1.36 [0.97 - 1.93], p = 0.082
cys-gly, oxidized	1.04 [0.74 - 1.44], p = 0.808	1.13 [0.8 - 1.55], p = 0.478	0.93 [0.66 - 1.29], p = 0.693	1.01 [0.71 - 1.42], p = 0.933
cysteinylglycine disulfide*	1.05 [0.78 - 1.54], p = 0.788	1.31 [0.93 - 1.8], p = 0.113	1.25 [0.88 - 1.78], p = 0.220	1.22 [0.87 - 1.68], p = 0.248
ergothioneine	0.85 [0.59 - 1.23], p = 0.382	0.99 [0.68 - 1.46], p = 0.940	1.22 [0.86 - 1.72], p = 0.263	1.31 [0.9 - 1.91], p = 0.161
gentisate	0.87 [0.6 - 1.24], p = 0.438	1.1 [0.75 - 1.6], p = 0.613	1.08 [0.78 - 1.48], p = 0.631	0.63 [0.44 - 0.89], p = 0.009
glutamate	1.01 [0.71 - 1.41], p = 0.957	1.24 [0.86 - 1.79], p = 0.240	1.25 [0.91 - 1.69], p = 0.153	1.51 [1.09 - 2.12], p = 0.015
glycerol	1.24 [0.87 - 1.81], p = 0.254	1.42 [0.95 - 2.19], p = 0.105	1.26 [0.9 - 1.8], p = 0.185	1.99 [1.33 - 3.07], p = 0.001
glycosyl-N-(2-hydroxynervonoyl)-sphingosine (d18:1/24:1(2OH))*	1.11 [0.79 - 1.58], p = 0.543	1.15 [0.8 - 1.73], p = 0.488	1.16 [0.83 - 1.65], p = 0.389	1.45 [0.99 - 2.23], p = 0.076

Maternal BMI Associated Metabolites in VDAART	Pre-eclampsia		Gestational Diabetes	
	**10-18 Weeks**	**32-38 Weeks**	**10-18 Weeks**	**32-38 Weeks**
hydroxyasparagine	1.22 [0.87 - 1.73], p = 0.248	2.14 [1.53 - 3], p = <0.001	1.26 [0.9 - 1.77], p = 0.178	1.15 [0.83 - 1.59], p = 0.386
mannonate*	1.16 [0.84 - 1.56], p = 0.336	1.67 [1.21 - 2.29], p = 0.002	1.33 [0.99 - 1.74], p = 0.046	1.84 [1.37 - 2.47], p = <0.001
mannose	1.23 [0.86 - 1.82], p = 0.280	1.06 [0.74 - 1.61], p = 0.751	1.6 [1.1 - 2.4], p = 0.018	1.26 [0.9 - 1.85], p = 0.203
metabolonic lactone sulfate	0.9 [0.63 - 1.27], p = 0.542	0.72 [0.48 - 1.05], p = 0.092	1.54 [1.11 - 2.14], p = 0.010	0.72 [0.51 - 1], p = 0.054
N-stearoyl-sphingosine (d18:1/18:0)*	1.2 [0.85 - 1.7], p = 0.293	1.52 [1.04 - 2.23], p = 0.031	1.41 [1.01 - 1.98], p = 0.042	2.09 [1.48 - 2.98], p = <0.001
pregnenetriol disulfate*	0.94 [0.65 - 1.35], p = 0.721	0.97 [0.67 - 1.45], p = 0.895	1.82 [1.28 - 2.63], p = 0.001	1.3 [0.92 - 1.85], p = 0.136
sphingomyelin (d17:2/16:0, d18:2/15:0)*	1.07 [0.75 - 1.53], p = 0.721	0.96 [0.66 - 1.43], p = 0.845	0.82 [0.6 - 1.15], p = 0.241	0.73 [0.53 - 1.02], p = 0.059
sphingomyelin (d18:0/18:0, d19:0/17:0)*	1.23 [0.86 - 1.73], p = 0.248	1.14 [0.79 - 1.65], p = 0.483	1.3 [0.93 - 1.82], p = 0.123	1.83 [1.3 - 2.62], p = <0.001
sphingomyelin (d18:1/18:1, d18:2/18:0)	1.5 [1.04 - 2.17], p = 0.031	1.94 [1.28 - 3], p = 0.002	0.72 [0.49 - 1.03], p = 0.077	1.4 [0.98 - 2.02], p = 0.070
sphingomyelin (d18:2/14:0, d18:1/14:1)*	1.15 [0.8 - 1.64], p = 0.452	0.95 [0.65 - 1.39], p = 0.793	0.68 [0.48 - 0.97], p = 0.035	0.43 [0.3 - 0.61], p = <0.001
sphingomyelin (d18:2/16:0, d18:1/16:1)*	1.2 [0.86 - 1.69], p = 0.288	1.26 [0.88 - 1.85], p = 0.220	0.68 [0.48 - 0.96], p = 0.028	0.89 [0.65 - 1.25], p = 0.499
sphingomyelin (d18:2/18:1)*	1.43 [1 - 2.03], p = 0.047	1.16 [0.8 - 1.71], p = 0.448	0.65 [0.46 - 0.92], p = 0.014	1.06 [0.76 - 1.5], p = 0.753
sphingomyelin (d18:2/21:0, d16:2/23:0)*	1.16 [0.83 - 1.63], p = 0.384	1.11 [0.77 - 1.61], p = 0.581	0.64 [0.45 - 0.89], p = 0.009	0.75 [0.55 - 1.04], p = 0.081
tartronate (hydroxymalonate)	0.98 [0.71 - 1.39], p = 0.882	0.76 [0.55 - 1.06], p = 0.094	0.93 [0.67 - 1.34], p = 0.679	0.65 [0.48 - 0.88], p = 0.005

Supplementary Table 6. Associations between maternal BMI-associated metabolites and gestational diabetes or preeclampsia in VDAART.

Odds ratios and 95% confidence intervals from multivariable logistic regression models for each of the 46 BMI-associated maternal serum metabolites in relation to gestational diabetes and preeclampsia at 10–18 weeks and 32–38 weeks of gestation. Models were adjusted for social circumstances (the first

principal component of household income, maternal education level, and maternal age at birth), child sex, smoking during pregnancy and self-reported ethnicit. Significant associations ($p < 0.05$) are highlighted in bold in the table, of note these analyses are hypothesis-generating and presented without multiple testing correction. P-values are from two-sided Wald tests.

COPSAC2010 Pregnancy Complications	Maternal BMI 24 Week Metabolite Score (Adjusted for Maternal BMI)	-
**Gestational Diabetes**	1.86 [0.87 - 4.09], p = 0.114)	-
**Preeclampsia**	1.62 [0.93 - 2.86], p = 0.093)	-
**Cesarean Section**	1.19 [0.91 - 1.55], p = 0.203)	-
**Emergency**	1.21 [0.87 - 1.7], p = 0.270)	-
**Elective**	1.12 [0.78 - 1.62], p = 0.547)	-
**Induction of Birth**	1.05 [0.84 - 1.32], p = 0.662)	-
**Antibiotics Administered to Mother at Birth**	1.11 [0.88 - 1.4], p = 0.385)	-
**VDAART Pregnancy Complications**	**Maternal BMI 10-18 Week Metabolite Score (Adjusted for Maternal BMI)**	**Maternal BMI 32-38 Week Metabolite Score (Adjusted for Maternal BMI)**
**Gestational Diabetes**	1.27 [0.82 - 1.96], p = 0.285)	1.91 [1.24 - 2.99], p = 0.004)
**Preeclampsia**	1.49 [0.93 - 2.39], p = 0.098)	2.12 [1.32 - 3.47], p = 0.002)
**Cesarean Section**	0.91 [0.73 - 1.14], p = 0.434)	0.98 [0.79 - 1.21], p = 0.817)
**Emergency**	0.94 [0.67 - 1.32], p = 0.718)	0.9 [0.65 - 1.25], p = 0.509)
**Elective**	0.93 [0.72 - 1.2], p = 0.594)	1.04 [0.82 - 1.32], p = 0.769)
**Induction of Birth**	1.09 [0.84 - 1.4], p = 0.532)	1.12 [0.88 - 1.43], p = 0.371)
**Antibiotics Administered to Mother at Birth**	1.06 [0.86 - 1.3], p = 0.575)	0.95 [0.79 - 1.16], p = 0.635)

Table S7. The association of the maternal BMI metabolite score at 10-18 weeks (early) and 32-38 weeks (late) gestational period, and pregnancy complications, in multivariable modelling further adjusted for maternal BMI. Odds ratios (per 1 SD change of metabolite score) and 95% confidence intervals from multivariable logistic regression models. P-values are from two-sided Wald tests. No adjustments were made for multiple comparisons.

SUPPLEMENTARY CODE

Section1: Deriving the 46-metabolites using overlapping metabolites from the COPSAC2010 and VDAART cohorts.

—

```
set.seed(123)

setwd("//192.168.1.59/Research/metabolicsyn/Diet, COPSYCH, ADHD/Revision/Data")

load("data.Rdata")

Week24
<-rio::import('//192.168.1.59/Data/COPSAC-2010/Bloodsamples/ABC0225_Mother_w24_Metabolites.
xlsx', sheet=2)  %>% select(-2,-3)

missing_percentages <- sapply(Week24, function(col) {

  sum(is.na(col)) / length(col) * 100

})

# Create a new dataframe with metabolite names and missingness percentages

metabolite_data <- data.frame(

  Metabolite = names(Week24),

  Missingness_Percentage = missing_percentages

)

metabolite_data <- metabolite_data %>% filter(Missingness_Percentage<33)

selected_columns_24w <- Week24 %>%

  select(all_of(metabolite_data$Metabolite))
```

```
### IMPUTING MISSING DATA USING RF

missing_values <- colSums(is.na(selected_columns_24w))

print(missing_values)

selected_columns_24w <- selected_columns_24w %>% select(-ABCNO)

selected_columns_24w <- selected_columns_24w %>%

  select(-starts_with("XX"))

#rf_imputed_24w_removedXX <- missForest(selected_columns_24w, verbose = TRUE)## TAKES > 1 HOUR TO IMPUTE, LOAD IMPUTED BELOW

setwd("//192.168.1.59/Research/metabolicsyn/Diet, COPSYCH, ADHD/Revision/RF_Imputed")

#save(file = 'rf_imputed_24w_removedXX.Rdata', list = c('rf_imputed_24w_removedXX'))

load("rf_imputed_24w_removedXX.Rdata")

###

Week24 <- cbind(Week24 %>% select(ABCNO), rf_imputed_24w_removedXX$ximp)

logtransform <- function(x, na.rm = FALSE) (x %>% log())

Week24<-Week24 %>% dplyr::mutate(across(starts_with("X"), logtransform))

scale2 <- function(x, na.rm = FALSE) (x %>% scale())

Week24 <- Week24 %>% dplyr::mutate(across(starts_with("X"), scale2))

m <- Week24

m_for_overlap<-m ### SAVE HERE TO USE FOR LATER WITH THE OTHER 9 METABOLOME TIMEPOINTS
```

```
setwd("//192.168.1.59/UserFolders/david.horner/VDAART")

week10_VDAART<-read.csv("VDAART_M1_raw_mets.csv")

week10_VDAART<- week10_VDAART %>% select(-1:-3, -5)

VV32<- read_xlsx("Metabolite_Info_Plasma_VDAART_10.18.GW (1).xlsx")

missing_percentages <- sapply(week10_VDAART, function(col) {

  sum(is.na(col)) / length(col) * 100

})

metabolite_data <- data.frame(

  Metabolite = names(week10_VDAART),

  Missingness_Percentage = missing_percentages

)

metabolite_data <- metabolite_data %>% filter(Missingness_Percentage<33)

selected_columns_week10_VDAART <- week10_VDAART %>%

  select(all_of(metabolite_data$Metabolite))

selected_columns_week10_VDAART <- selected_columns_week10_VDAART %>% select(-vid)

#rf_imputed_10w<- missForest(selected_columns_week10_VDAART, verbose = TRUE)## TAKES >
1 HOUR TO IMPUTE, LOAD IMPUTED BELOW

setwd("//192.168.1.59/Research/metabolicsyn/Diet, COPSYCH, ADHD/Revision/RF_Imputed")

#save(file = 'rf_imputed_10w.Rdata', list = c('rf_imputed_10w'))

load("rf_imputed_10w.Rdata")
```

```
Week10 <- cbind(week10_VDAART %>% select(vid), rf_imputed_10w$ximp)

V32_imputed<-Week10

V32_imputed <- V32_imputed %>% dplyr::mutate(across(starts_with("X"), logtransform))

V32_imputed <- V32_imputed %>% dplyr::mutate(across(starts_with("X"), scale2))

mm<-read.csv("//192.168.1.59/UserFolders/min.kim/xDavid/Metabolite_Info_w24w1m18.csv")

m_trial <- m_for_overlap %>% pivot_longer(!ABCNO, names_to = "Metabolite", values_to = "value")

Mother_24w_COMP_ID <- left_join(m_trial, mm %>% select(PATHWAY_SORTORDER, COMP_ID, BIOCHEMICAL), by = c("Metabolite"="PATHWAY_SORTORDER")) %>% mutate(TIME="Mother_24w")

V32<-V32_imputed

n_trial <- V32 %>% pivot_longer(!vid, names_to = "Metabolite", values_to = "value")

###

Mother_VDAART_child_COMP_ID <- left_join(n_trial, VV32 %>% select(PATHWAY_SORTORDER, COMP_ID, BIOCHEMICAL), by = c("Metabolite"="COMP_ID")) %>%mutate(TIME="VDAART32")

hold_thing <- Mother_VDAART_child_COMP_ID %>% pivot_wider(vid,names_from = BIOCHEMICAL, values_from = value) %>% ungroup()

names_VDAART <- colnames(hold_thing[,-1])

m_data <- Mother_24w_COMP_ID %>%

    filter(BIOCHEMICAL %in% names_VDAART) %>% pivot_wider(ABCNO,names_from = BIOCHEMICAL, values_from = value) %>% ungroup()

hold_thing2 <- Mother_24w_COMP_ID %>% pivot_wider(ABCNO,names_from = BIOCHEMICAL, values_from = value) %>% ungroup()
```

```
names_COPSAC <- colnames(hold_thing2[,-1])

n_data <- Mother_VDAART_child_COMP_ID %>%

  filter(BIOCHEMICAL %in% names_COPSAC) %>% pivot_wider(vid,names_from = BIOCHEMICAL, values_from = value) %>% ungroup()

n_data_10weeks <- n_data

## VISUAL CHECK OF DATA BETWEEN COHORTS

#ic <- intersect(colnames(m_data),colnames(n_data %>% dplyr::rename(ABCNO=vid)))

#DBS <- bind_rows(m_data %>% dplyr::select(all_of(ic)),n_data %>% dplyr::rename(ABCNO=vid)%>%  dplyr::select(all_of(ic)))

#m <- prcomp(DBS[,-1], scale. = T)

#dfPCA<- data.frame(DBS,m$x)

#dfPCA %>%

#  ggplot(data = .,aes(PC1,PC2, color = ABCNO>3000)) + geom_point() + facet_wrap(~(ABCNO>3000))

###

XX <- m_data %>% ungroup() %>% select(-1)

hold <- left_join(m_data, data %>% select(ABCNO, Mother_BMI), by ="ABCNO") %>% ungroup() %>%  select(-1)

XX2 <- XX

XX2$Mother_BMI <- hold$Mother_BMI

XX2 <- XX2 %>% filter(!is.na(Mother_BMI))
```

```
XX2 <- XX2 %>% mutate(Mother_BMI=scale(Mother_BMI))

repCV10 <- trainControl(method = "repeatedcv",

            number = 5,

            repeats = 10,

            returnResamp = "all",

            savePredictions = "all",

            allowParallel = T,

            verboseIter = F)

SPLS_model_2 <- train(Mother_BMI ~ ., data = XX2,

           method = 'spls',

           preProc = c("center", 'scale'),

           tuneGrid = expand.grid(K = 1, # K sets number of components

                      eta = seq(0,1,0.1), # eta sets sparsity

                      kappa = 0.5),

           trControl = repCV10)

plot(SPLS_model_2)

SPLS_model_2 $results$RMSE

best_model_row <- which.min(SPLS_model_2 $results$RMSE)

best_model_rmse <- SPLS_model_2$results$RMSE[best_model_row]
```

```
rmse_threshold <- 0.01

candidate_rows <- which(abs(SPLS_model_2 $results$RMSE - best_model_rmse) <= rmse_threshold)

best_eta_row <- which.max(SPLS_model_2 $results$eta[candidate_rows])

best_eta <- SPLS_model_2 $results$eta[candidate_rows][best_eta_row]

SPLS_model_2_retrained <- train(Mother_BMI ~ ., data = XX2,

                    method = 'spls',

                    preProc = c("center", 'scale'),

                    tuneGrid = expand.grid(K = 1, # Use the best K value

                              eta = best_eta, # Use the best eta value

                              kappa = 0.5),

                    trControl = repCV10)

SPLS_model_2$finalModel$projection %>% data.frame() %>% rownames_to_column(var = 'Feature')

SPLS_model_2_retrained$finalModel$projection %>% data.frame() %>% rownames_to_column(var = 'Feature')

XX2

COPSAC2010_10weekmbmi<-cbind(m_data %>% select(1), predict(SPLS_model_2,newdata=m_data)) %>% dplyr::rename(mbmi_24w10="predict(SPLS_model_2, newdata = m_data)") %>% mutate(mbmi_24w10=scale(mbmi_24w10))
```

—

Section2: Code for the Systematic backward elimination strategy Mediation Analysis (Gestation Diabetes example)

```
library(mediation)

set.seed(123)

setwd("/home/david/mbmi_birth/Data")

load("data.Rdata")

glimpse(data)

data<- left_join(data, yhat_24w, by = "ABCNO")

loads_VDAART_overlap <- SPLS_model_2_retrained$finalModel$projection %>% data.frame() %>%
rownames_to_column(var = 'Feature')

glimpse(m_data)

glimpse(loads_VDAART_overlap$Feature)

loads_VDAART_overlap$Feature <- gsub("`", "", loads_VDAART_overlap$Feature)

metabolites_18 <- m_data %>% select(ABCNO, loads_VDAART_overlap$Feature)

data <- left_join(data, metabolites_18, by = "ABCNO")

colnames(data)

setwd("/home/david/mbmi_birth/Data")

data <- left_join(data, read_xlsx("gestdiabetes.xlsx"), by = "ABCNO")

data <- data %>% mutate(gestdiabetes=as.factor(gestdiabetes))
```

```
X <- data

X <- X %>% filter(!is.na(Mother_BMI))

X <- X %>% filter(!is.na(gestdiabetes))

X <- X %>% filter(!is.na(yhat_24w))

trial<-X%>%select(`16a-hydroxy DHEA 3-sulfate`:`tartronate (hydroxymalonate)`)

extract_mediation_summary <- function (x) {


  clp <- 100 * x$conf.level

  isLinear.y <- ((class(x$model.y)[1] %in% c("lm", "rq")) ||

                 (inherits(x$model.y, "glm") && x$model.y$family$family ==

                   "gaussian" && x$model.y$family$link == "identity") ||

                 (inherits(x$model.y, "survreg") && x$model.y$dist ==

                   "gaussian"))


  printone <- !x$INT && isLinear.y


  if (printone) {


        smat <- c(x$d1, x$d1.ci, x$d1.p)

        smat <- rbind(smat, c(x$z0, x$z0.ci, x$z0.p))
```

```
        smat <- rbind(smat, c(x$tau.coef, x$tau.ci, x$tau.p))
        smat <- rbind(smat, c(x$n0, x$n0.ci, x$n0.p))

        rownames(smat) <- c("ACME", "ADE", "Total Effect", "Prop. Mediated")

    } else {
        smat <- c(x$d0, x$d0.ci, x$d0.p)
        smat <- rbind(smat, c(x$d1, x$d1.ci, x$d1.p))
        smat <- rbind(smat, c(x$z0, x$z0.ci, x$z0.p))
        smat <- rbind(smat, c(x$z1, x$z1.ci, x$z1.p))
        smat <- rbind(smat, c(x$tau.coef, x$tau.ci, x$tau.p))
        smat <- rbind(smat, c(x$n0, x$n0.ci, x$n0.p))
        smat <- rbind(smat, c(x$n1, x$n1.ci, x$n1.p))
        smat <- rbind(smat, c(x$d.avg, x$d.avg.ci, x$d.avg.p))
        smat <- rbind(smat, c(x$z.avg, x$z.avg.ci, x$z.avg.p))
        smat <- rbind(smat, c(x$n.avg, x$n.avg.ci, x$n.avg.p))

        rownames(smat) <- c("ACME (control)", "ACME (treated)",
            "ADE (control)", "ADE (treated)", "Total Effect",
            "Prop. Mediated (control)", "Prop. Mediated (treated)",
```

```
                    "ACME (average)", "ADE (average)", "Prop. Mediated (average)")

  }

  colnames(smat) <- c("Estimate", paste(clp, "% CI Lower", sep = ""),
                      paste(clp, "% CI Upper", sep = ""), "p-value")
  smat

}
oneloop <- function(x,xmet,load){
  RES_backwardelim <- data.frame()
  # kill one variable (0 is not to kill any)
  for (i in 0:length(load)){
        # lds <- as.vector(loads$Comp.1)
        lds <- load
        # print(i)
        lds[i] <- 0
        x$comp <- xmet %*% lds
        # x %>% ggplot(data = ., aes(comp,yhat_24w)) + geom_point()
```

```
        # valuate model
        mod1 = lm(comp ~ Mother_BMI +as.factor(Sex)  + Socialcircumstances  +  AllSmoking + Diet_PC1 + Diet_PC2 + Diet_PC3, data=x)
        mod2 = glm(gestdiabetes ~ Mother_BMI + comp + as.factor(Sex)  + Socialcircumstances  + AllSmoking + Diet_PC1 + Diet_PC2 + Diet_PC3, data=x, family="binomial")
        Med <- mediation::mediate(mod1,mod2, treat="Mother_BMI",mediator="comp", sims = 10000)
  z<-extract_mediation_summary(summary(Med)) %>% as.data.frame() %>%
   rownames_to_column('Mediation') %>%
   mutate(variabledeletion = i)


        RES_backwardelim <- RES_backwardelim %>% bind_rows(z)
 }
 killthis <- RES_backwardelim %>%
        filter(Mediation=='ACME (average)') %>%
        arrange(-Estimate) %>%
        head(1)
 if (killthis$variabledeletion>0) {
  killthis$variabledeletionname <- colnames(xmet)[killthis$variabledeletion]
 } else {killthis$variabledeletionname <- 'NN'}
 print(killthis)
```

```
  return(killthis)

}

x <- X

colnames(x)

xmet <- as.matrix(x[,138:183])

load <- loads_VDAART_overlap$Comp.1

r <- oneloop(x,xmet,load)

j <- r$variabledeletion

RES <- r

c <- 0

while (j!=0){

  c <- c + 1

  xmet <- xmet[,-j]

  load <- load[-j]

  r <- oneloop(x,xmet,load) %>% mutate(iteration = c)

  j <- r$variabledeletion

  RES <- RES %>% bind_rows(r)

}

RES

xmet <- as.matrix(x[,138:183])
```

```
ic <- !(colnames(xmet) %in% RES$variabledeletionname)

gestdiabetes_loads_VDAART <- loads_VDAART_overlap %>% filter(!Feature %in% RES$variabledeletionname)

x$comp <- as.matrix(xmet[,ic]) %*% loads_VDAART_overlap$Comp.1[ic]

# valuate model

mod1 = lm(comp ~ Mother_BMI +as.factor(Sex) + Socialcircumstances + AllSmoking + Diet_PC1 + Diet_PC2 + Diet_PC3, data=x)

mod2 = glm(gestdiabetes ~ Mother_BMI + comp +as.factor(Sex) + Socialcircumstances + AllSmoking + Diet_PC1 + Diet_PC2 + Diet_PC3, data=x, family="binomial")

Med_VDAART <- mediation::mediate(mod1,mod2, treat="Mother_BMI",mediator="comp", sims =10000)

gestdiabetes_results_VDAART<-extract_mediation_summary(summary(Med_VDAART)) %>% as.data.frame() %>%
  rownames_to_column('Mediation')

setwd("/home/david/mbmi_birth/Data")

write.csv(gestdiabetes_loads_VDAART, file = "gestdiabetes_loads_VDAART.csv")

write.csv(gestdiabetes_results_VDAART, file = "gestdiabetes_results_VDAART.csv")
```
