# Supplementary material for: A metabolomic signature of maternal BMI is associated with pregnancy complications across two independent pregnancy cohorts
Source: Commun Med (Lond). 2025 Dec 17;6:38. doi: 10.1038/s43856-025-01289-5 (PMC12820178; doi:10.1038/s43856-025-01289-5)
Supplement: Supplementary file 3 — Description of Additional Supplementary files [file 43856_2025_1289_MOESM3_ESM.docx]

**Description of Additional Supplementary Files**

File name: Supplementary Data

Description: Dataset containing the loading for the 46 metabolites selected by sparse partial least squares modelling in COPSAC2010 at 24 weeks gestation. For each metabolite, the file provides its loading (metabolite score), biochemical pathway annotations, rank order, and mediation status for the maternal BMI-gestational diabetes association. These data correspond to the metabolite scores visualised in Figure 1.
